# Body image and its influencing factors in colorectal cancer patients with a stoma: a latent profile analysis

**DOI:** 10.3389/fpubh.2026.1860267

**Published:** 2026-06-08

**Authors:** Xinyue Tian, Rongrong Ma, Yongxia Chen, Yudie Shao, Huiru Ma, Min Dong, Rui Huo, Binbin Wang

**Affiliations:** 1 The First Affiliated Hospital of Bengbu Medical University, Bengbu, China; 2 School of Nursing, Bengbu Medical University, Bengbu, China

**Keywords:** body image, colorectal cancer, influencing factors, latent profile, stoma

## Abstract

**Aim:**

To identify the underlying profiles of postoperative body image in patients with colorectal cancer who have undergone stoma surgery and to analyze the factors influencing these profiles, thereby providing a basis for developing targeted intervention strategies.

**Methods:**

A convenience sample of 349 colorectal cancer patients with a stoma receiving treatment at a Grade A tertiary hospital in Anhui Province was selected as the study population. Data were collected using a general questionnaire, the Body Image Scale, the Perceived Social Support Scale, the Hospital Anxiety and Depression Scale, the Ostomy Adjustment Inventory-23, and the Brief Illness Perception Questionnaire. Latent profile analysis was used to identify latent profiles of body image, and univariate and multivariate logistic regression were employed to explore their influencing factors.

**Results:**

A total of 332 valid questionnaires were collected in this study, representing a response rate of 95.1%. Body image among colorectal cancer patients with a stoma can be classified into three subtypes: low body image disturbance-emotional stability group (31.7%), moderate body image disturbance-cognitive regulation group (39.4%), and high body image disturbance-social avoidance group (28.9%). Age, gender, place of residence, educational level, stoma complications, time since stoma creation, social support, anxiety and depression, stoma adaptation, and illness perception were identified as factors influencing the latent profiles of body image among colorectal cancer patients with a stoma.

**Conclusion:**

The study findings suggest that body image among patients with colorectal cancer who have a stoma exhibits significant heterogeneity within the population. Healthcare professionals can identify patients with different body image profiles at an early stage and develop targeted nursing interventions to alleviate body image disturbances and promote the restoration of social functioning and reintegration.

## 1 Introduction

According to global cancer statistics for 2022, colorectal cancer ranked third in new cases and second in deaths among all cancers (1). Currently, surgery is the primary treatment for colorectal cancer (2), with 50 to 60% of patients requiring a stoma (3). Because a stoma alters a patient’s body structure and bowel habits, patients must wear a stoma bag for the long-term and engage in continuous self-management. Concerns about visible appearance, odor, and limitations in intimate relationships can easily lead to a sense of compromised bodily integrity (4). Furthermore, influenced by traditional Chinese cultural values that emphasize bodily integrity and social acceptance, patients with a stoma are more prone to body image disturbance. As survival rates for colorectal cancer patients continue to rise, body image has become one of the key factors affecting their long-term recovery (5).

Body image (BI) refers to an individual’s subjective perception and evaluation of their physical appearance and function (6). Body image disturbance (BID) occurs when an individual experiences negative cognitive, emotional, or behavioral reactions regarding their physical appearance, function, or overall image (7). Among colorectal cancer patients who have undergone ostomy surgery (8), the establishment of a stoma leads to significant physical changes that profoundly affect a person’s sense of identity, attractiveness, and sexual confidence. These changes in BI persist from the initial diagnosis through the entire course of treatment and survival. Studies have shown that BID is prevalent among colorectal cancer patients with a stoma, affecting nearly half of patients and contributing to reduced self-confidence, psychological distress, and impaired quality of life (9, 10).

Current research on BI among colorectal cancer patients with a stoma indicates (11) that patients are prone to developing negative BI and social avoidance behaviors when facing changes in physical appearance and function. As research has progressed, studies on BI have gradually shifted from examining its manifestations to exploring its influencing factors. Factors such as social support (12), anxiety and depression (13), stoma adaptation (14), illness perception (15), and sociodemographic and clinical characteristics (16) may all influence patients’ BI. However, existing studies predominantly employ variable-centered approaches, treating patients as a homogeneous group, thereby overlooking the variability in BI among individuals. Therefore, it is necessary to adopt an individual-differences perspective to further explore the potential heterogeneity of BI among colorectal cancer patients with a stoma and their associated influencing factors.

Latent profile analysis (LPA) (17) is an individual-centered analytical method that identifies and classifies individuals into distinct latent profiles based on their shared characteristics. Unlike traditional binary classification based on BIS total score thresholds (e.g., BIS ≥ 10) (18), LPA not only identifies different latent profiles of BI but also analyzes the corresponding sociodemographic characteristics, clinical features, and influencing factors of each profile. The objectives of this study are (1) to explore the characteristics of latent profiles of BI among colorectal cancer patients with a stoma, and (2) to determine the influence of sociodemographic, clinical, and psychosocial factors on different latent profiles of BI. This will assist healthcare professionals in developing intervention strategies to improve patients’ BI and quality of life.

## 2 Methods

### 2.1 Sampling and eligibility criteria

Convenience sampling was used to select colorectal cancer patients with a stoma who were undergoing follow-up at the Department of Gastrointestinal Surgery and the Wound and Ostomy Clinic of a Grade A tertiary hospital in Anhui Province between March 2025 and March 2026. Inclusion criteria: ① Age ≥ 18 years; ② Pathologically diagnosed with colorectal cancer; ③ ≥1 month since the first ostomy surgery; ④ Clear consciousness and good communication ability. Exclusion criteria: ① Concurrent failure of vital organ function or cancer recurrence/metastasis; ② Diagnosed with severe psychiatric disorders that could impair cognitive function or questionnaire completion; ③ Presence of two or more stomas after surgery; ④ Experience of a major traumatic event. Based on Kendall’s (19) principle that the sample size should be 10 to 20 times the number of independent variables, 21 independent variables were initially identified. After accounting for a 20% non-response rate, the minimum required sample size was 263 participants. Ultimately, 332 colorectal cancer patients with a stoma were included in the study.

### 2.2 Data collection

Before the survey began, two nursing graduate students were trained. Starting in March 2025, patients meeting the inclusion and exclusion criteria were selected from the hospital’s outpatient clinics and inpatient wards. Researchers personally distributed the questionnaires, which participants completed on-site and returned immediately. For patients who had difficulty completing the questionnaire, such as those who were older or had lower educational levels, researchers provided assistance. During the survey, researchers were available to answer any questions participants had to ensure their understanding and to improve the accuracy and completeness of their responses. All participants voluntarily agreed to take part in the study, signed informed consent forms, and were formally informed of their rights regarding the study, including the right to withdraw.

### 2.3 Ethical considerations

All participants voluntarily agreed to participate and signed informed consent forms. This study was approved by the Ethics Committee of the First Affiliated Hospital of Bengbu Medical University (No. [2024]KY017).

### 2.4 Measures

#### 2.4.1 Demographic questionnaire

The research team designed a demographic questionnaire, which included questions on age, gender, average monthly household income, place of residence, educational level, cancer stage, time since stoma creation, type of stoma, stoma complications, and adjuvant therapy.

#### 2.4.2 Body image scale

The Body Image Scale (BIS) was developed by Hopwood et al. (20) and adapted into Chinese by Song et al. (21) to specifically assess BI disturbance in patients with rectal cancer. The scale comprises two dimensions: body perception and appearance perception. It employs a 4-point Likert scale ranging from “not at all” to “very much,” with scores ranging from 0 to 3. The total score ranges from 0 to 30, with higher scores indicating poorer BI. The Cronbach’s *α* coefficient for the scale is 0.92; in this study, the Cronbach’s α coefficient was 0.939.

#### 2.4.3 Perceived social support scale

The Perceived Social Support Scale (PSSS), developed by Zimet et al. (22) and adapted into Chinese by Zhang et al. (23), is used to measure individuals’ perceptions of social support from family, friends, and other significant others. The scale consists of 12 items across three dimensions: family support, friend support, and other support. It employs a 7-point Likert scale from “strongly disagree” to “strongly agree,” with a total score of 12–84. A higher score indicates that the patient receives greater social support. Cronbach’s *α* ranges from 0.813 to 0.840; in this study, the Cronbach’s α for this scale was 0.868.

#### 2.4.4 Hospital anxiety and depression scale

The Hospital Anxiety and Depression Scale (HADS) was developed by Zigmond et al. (24) and adapted into Chinese by Ye et al. (25). It is used to assess common symptoms of anxiety and depression in non-psychiatric inpatients and outpatients. The scale comprises two subscales, anxiety and depression, each containing seven items, for a total of 14 items. Each item is scored from 0 to 3, with higher scores indicating more severe symptoms. A score >8 is considered indicative of anxiety or depressive symptoms (26). The overall Cronbach’s *α* coefficient is 0.85; in this study, the overall Cronbach’s α coefficient for this scale was 0.91.

#### 2.4.5 Ostomy adjustment inventory-23

The Ostomy Adjustment Inventory-23 (OAI-23), developed by Simmons et al. (27) and adapted into Chinese by Gao et al. (28), is used to assess adjustment among stoma patients in a Chinese cultural context. The inventory comprises three dimensions: persistent worry, positive attitude toward life, and acceptance, and it employs a 5-point Likert scale. The scale ranges from 0 to 80, with higher scores indicating greater ostomy adjustment. The overall Cronbach’s alpha coefficient for this scale is 0.886; in this study, the Cronbach’s *α* coefficient was 0.90.

#### 2.4.6 Brief illness perception questionnaire

The Brief Illness Perception Questionnaire (BIPQ) was developed by Broadbent et al. (29) and translated into Chinese by Mei et al. (30) to assess patients’ perceptions of their illness and emotional characteristics. The measure comprises three dimensions: cognitive representations, emotional representations, and knowledge about the illness, with a total of nine items. Except for the item regarding the cause of the illness, all other items are scored on a 0–10 scale, with a total score ranging from 0 to 80. A higher score indicates a more negative illness perception. The Chinese version of the scale has a Cronbach’s *α* coefficient of 0.77, and in this study, the Cronbach’s α coefficient for this scale was 0.778.

### 2.5 Statistical methods

Data analysis was performed using SPSS 26.0 and Mplus 8.3 software. Latent profile analysis was conducted using the 10-item BIS scale scores as observed variables to identify latent profiles of body image among colorectal cancer patients with a stoma. The initial assumption was that a single category existed. Subsequently, the number of categories was gradually increased, and the fit indices of each model were compared. We selected the optimal model based on fit indices. Fit indices for latent profile models fall into three categories: (1) Model fit tests, including the Akaike Information Criterion (AIC), the Bayesian Information Criterion (BIC), and the sample-corrected Bayesian Information Criterion (aBIC). Smaller values for these indices indicate better model fit. (2) Classification accuracy was assessed using entropy values ranging from 0 to 1. The closer the entropy value is to 1, the higher the classification accuracy. (3) Likelihood ratio test metrics, including the Lo–Mendell–Rubin likelihood ratio (LMR) and the bootstrap-based likelihood ratio test (BLRT), are used to compare model fit between adjacent models. A *p*-value <0.05 indicates a significant difference between the K-class models. Normally distributed continuous variables are expressed as mean ± standard deviation (*x* ± *s*), whereas non-normally distributed variables are expressed as median and interquartile range [*M* (P25, P75)]. Categorical data are presented as case counts, percentages, or proportions. Chi-square tests and the Kruskal–Wallis *H* test were used to compare differences in demographic and clinical characteristics among the latent profiles. The classification results from the latent profile analysis were used as the dependent variable. During the variable selection process, variables with statistical significance were included in the multivariate model based on clinical relevance and univariate results. Multivariate logistic regression was used to analyze the factors influencing different latent profiles of BI in colorectal cancer patients with a stoma. A *p*-value < 0.05 was considered statistically significant.

## 3 Results

### 3.1 Participants

A total of 349 questionnaires were distributed, and 332 valid responses were received, resulting in a response rate of 95.1%. Reasons for withdrawal included physical discomfort, refusal for personal reasons, and time constraints.

### 3.2 Sociodemographic and clinical characteristics

The mean age of the respondents was 60.66 years (SD = 12.742; range 29–88). Among the 332 participants, 16.3% held a college education or higher. A significant proportion of participants (59.0%) came from rural areas, and 54.2% reported household incomes below 3,000 yuan.

Regarding clinical variables, 99 patients had undergone ostomy surgery within the past 3 months, Patients with Stage I–II disease accounted for 46.4% of the sample. A total of 44.9% of patients received adjuvant therapy; more than half (50.3%) had a temporary stoma, and some participants (35.2%) experienced stoma complications (see Table 1).

**Table 1:** Univariate analysis of general characteristics and potential body image profiles in colorectal cancer patients with a stoma (*n* = 332).

Variables	Total (*n* = 332)	Low body image disturbance-emotional stability group (*n* = 105)	Moderate body image disturbance-cognitive regulation group (*n* = 131)	High body image disturbance-social avoidance group (*n* = 96)	Statistical values	*P*
Age, in years					6.434^b^	0.040
<50	70 (21.1)	22 (21.2)	20 (15.2)	28 (29.2)		
≥50	262 (78.9)	83 (79.0)	111 (84.7)	68 (70.8)		
Gender					17.131^b^	<0.001
Male	203 (61.1)	78 (74.3)	81 (61.8)	44 (45.8)		
Female	129 (38.9)	27 (25.7)	50 (37.9)	52 (54.2)		
Average monthly household income per capita, in RMB					31.598^a^	<0.001
<3,000	178 (53.6)	34 (32.4)	79 (60.3)	65 (67.7)		
3,000-4,999	97 (29.2)	40 (38.1)	36 (27.5)	21 (21.9)		
≥5,000	57 (17.2)	31 (29.5)	16 (12.2)	10 (10.4)		
Educational level					29.594^a^	<0.001
Elementary school and below	68 (20.5)	15 (14.3)	37 (28.2)	16 (16.7)		
Junior high school	122 (36.7)	20 (19.0)	47 (35.9)	55 (57.3)		
High school and vocational school	88 (26.5)	39 (37.1)	33 (25.2)	16 (16.7)		
College and above	54 (16.3)	31 (29.5)	14 (10.7)	9 (9.4)		
Place of residence					64.834^b^	<0.001
Rural	196 (59.0)	29 (27.6)	91 (69.5)	76 (79.2)		
Urban	136 (41.0)	76 (72.4)	40 (30.5)	20 (20.8)		
Cancer stage					25.009^a^	<0.001
Stage I–II	154 (46.4)	59 (56.2)	66 (50.4)	29 (30.2)		
Stage III	112 (33.7)	39 (37.1)	41 (31.3)	32 (33.3)		
Stage IV	66 (19.9)	7 (6.7)	24 (18.3)	35 (36.5)		
Time since stoma creation					26.849^b^	<0.001
≤3 months	99 (29.8)	14 (13.3)	58 (44.7)	27 (28.1)		
>3 months	233 (70.2)	91 (86.7)	73 (55.3)	69 (71.9)		
Stoma type					1.870^b^	0.413
Temporary stoma	167 (50.3)	57 (54.3)	67 (51.1)	43 (44.8)		
Permanent stoma	165 (49.7)	48 (45.7)	64 (48.9)	53 (55.2)		
Stoma complications					40.444^b^	<0.001
Yes	117 (35.2)	20 (19.0)	39 (29.5)	58 (60.4)		
None	215 (64.8)	85 (81.0)	92 (70.2)	38 (39.6)		
Adjuvant therapy					32.599^b^	<0.001
Yes	149 (44.9)	28 (26.7)	57 (43.5)	64 (66.7)		
None	183 (55.1)	77 (73.3)	74 (56.5)	32 (33.3)		
Perceived social support	62.0 (55, 69)	68.0 (61.0,71.75)	62.0 (57.0, 68.0)	55.0 (49.0, 62.75)	64.190^a^	<0.001
Anxiety and depression	9.0 (6.00,14.00)	6.0 (4.00, 8.00)	9.0 (6.00, 12.75)	15.5 (12.00, 20.00)	118.032^a^	<0.001
Ostomy adjustment	41.0 (33.00, 48.00)	48.5 (43.00, 53.00)	41.0 (36.00, 45.75)	30.0 (25.25, 37.75)	112.563^a^	<0.001
Illness perception	56.0 (49.00, 63.00)	50.5 (40.25, 59.00)	56.00 (51.00, 61.00)	60.0 (54.00, 65.00)	36.897^a^	<0.001

^a^*H*-value.

^b^*χ*^2^-value.

### 3.3 Latent profile model fit results

Five LPA models were fitted using scores from the 10 BIS items; the fit indices comparison is shown in Table 2. As the number of classes increased, the AIC, BIC, and aBIC values gradually decreased. The entropy values for all models exceeded the 0.8 threshold, indicating good classification accuracy. When the number of classes was two or three, both the LMR and BLRT values were statistically significant (*p* < 0.05). For the three-class model, the entropy reached a maximum of 0.943, and each latent profile accounted for more than 10% of participants, demonstrating good statistical stability and clinical interpretability.

**Table 2:** Fit indicators of the latent profile model of body image in colorectal cancer patients with a stoma (*n* = 332).

Model	AIC	BIC	aBIC	Entropy	*p*		Profile prevalence (%)
					LMR	BLRT	
1	9034.273	9110.376	9046.935	–	–	–	–
2	7460.458	7578.417	7480.083	0.923	<0.001	<0.001	0.556/0.445
3	6945.002	7104.818	6971.592	0.943	<0.001	<0.001	0.317/0.394/0.289
4	6818.352	7020.025	6851.906	0.926	0.2988	<0.001	0.311/0.351/0.235/0.103
5	6695.496	6939.025	6736.014	0.931	0.3752	<0.001	0.299/0.230/0.155/0.212/0.104

“–” indicates not applicable; aBIC, adjusted Bayesian information criterion; AI, Akaike information criterion; BIC, Bayesian information criterion; BLRT, bootstrap likelihood ratio test; LMR, Lo–Mendell–Rubin test.

### 3.4 Characteristics of the three latent profiles

**Figure 1:**
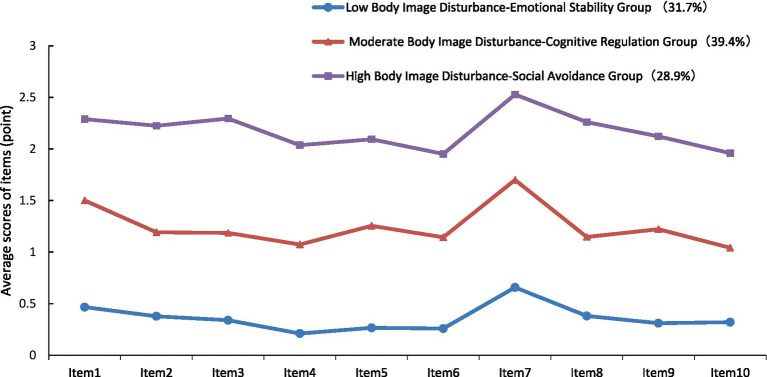
Characteristics of the three latent profiles of body image among colorectal cancer patients with a stoma.

After integrating fit indices and clinical relevance, the three-profile model was determined to be the optimal model for BI in colorectal cancer patients with a stoma. The item score distributions across the three profiles are shown in Figure 1. The first category comprised 31.7% of the participants. Scores on all items in this category were relatively low, suggesting that patients exhibited mild negative cognitive and emotional reactions to postoperative physical changes, and generally demonstrated good emotional stability. Therefore, it was named the “low body image disturbance-emotional stability group.” The second category comprised 39.4% of the participants. Item scores fell between those of the first and third categories, with all item scores at moderate levels. Participants in this group exhibited a certain degree of social avoidance in interpersonal interactions but still possessed some cognitive regulation and adaptive abilities. Therefore, it was designated as the “moderate body image disturbance-cognitive regulation group.” The third category comprised 28.9% of the participants. These participants scored high on all items, with the highest score on Item 7 (“Avoiding Crowds”) and notably high scores on Items 1, 3, and 8. This indicated significant distress regarding BI and bodily integrity, as well as a greater tendency toward social avoidance. Therefore, this category was named “high body image disturbance-social avoidance group.”

### 3.5 Univariate analysis of factors associated with latent profiles

Statistically significant differences were observed among the three latent profiles in terms of age, gender, place of residence, educational level, cancer stage, time since stoma creation, stoma complications, and adjuvant therapy. Furthermore, the Kruskal–Wallis *H* test revealed significant differences among the three latent profiles in perceived social support, anxiety and depression, ostomy adjustment, and illness perception scores (*p* < 0.001). Compared with the other two groups, patients in the high body image disturbance-social avoidance group reported lower perceived social support and ostomy adjustment scores, as well as higher anxiety and depression and illness perception scores. Detailed results are provided in Table 1.

### 3.6 Multivariate analysis of factors associated with latent profiles

Using the low body image disturbance-emotional stability group as the reference group, logistic regression was performed with the significant predictors identified in the univariate analysis; the results are presented in Table 3. Multicollinearity tests indicated that the VIF values for all independent variables were <5. Therefore, no significant multicollinearity was detected. The model fit results showed that the *p*-value for the likelihood ratio test was <0.001, indicating that the model was statistically significant. The results suggest that age, gender, place of residence, educational level, stoma complications, time since stoma creation, social support, anxiety and depression, ostomy adjustment, and illness perception were associated with latent profile membership among colorectal cancer patients with a stoma.

**Table 3:** Multivariate analysis of latent profiles of body image in colorectal cancer patients with a stoma (*n* = 332).

Variables	Moderate body image disturbance-cognitive regulation group				High body image disturbance-social avoidance group			
	*β*	*P*	OR	95% CI	*β*	*P*	OR	95% CI
Age, in years								
<50	0.293	0.541	1.340	0.525–3.420	2.127	0.001	8.391	2.351–29.949
Gender								
Male	−0.992	0.018	0.371	0.163–0.844	−1.728	0.001	0.178	0.062–0.509
Average monthly household income per capita, in RMB								
<3,000	0.296	0.618	1.344	0.420–4.305	−0.094	0.912	0.910	0.173–4.799
3,000-4,999	0.038	0.940	1.039	0.385–2.806	−1.408	0.087	0.245	0.049–1.225
Educational level								
Elementary school and below	0.991	0.162	2.693	0.672–10.788	0.468	0.665	1.597	0.192–13.268
Junior high school	1.408	0.020	4.089	1.251–13.364	2.556	0.006	12.885	2.074–80.033
High school and vocational school	0.721	0.163	2.056	0.747–5.656	1.101	0.201	3.007	0.556–16.274
Place of residence								
Rural	1.232	0.002	3.429	1.548–7.595	1.488	0.010	4.429	1.432–13.699
Cancer stage								
Stage I–II	−0.470	0.422	0.625	0.198–1.970	−1.220	0.088	0.295	0.073–1.200
Stage III	−0.789	0.204	0.454	0.134–1.536	−0.844	0.253	0.430	0.101–1.826
Time since stoma creation								
≤3 months	1.608	<0.001	4.995	2.172–11.485	1.194	0.034	3.302	1.095–9.957
Stoma complications								
Yes	0.462	0.310	1.588	0.651–3.875	1.772	0.002	5.881	1.916–18.048
Adjuvant therapy								
Yes	0.668	0.086	1.950	0.909–4.180	0.623	0.231	1.864	0.672–5.167
Perceived social support	−0.021	0.373	0.979	0.935–1.026	−0.061	0.041	0.941	0.888–0.998
Anxiety and depression	0.056	0.242	1.057	0.963–1.160	0.169	0.004	1.185	1.057–1.328
Ostomy adjustment	−0.046	0.037	0.955	0.915–0.997	−0.143	<0.001	0.867	0.814–0.922
Illness perception	0.043	0.016	1.043	1.008–1.080	0.056	0.033	1.058	1.004–1.114

Low body image disturbance-emotional stability group was used as the reference group.

## 4 Discussion

### 4.1 Significant heterogeneity in BI among colorectal cancer patients with a stoma

This study surveyed 332 colorectal cancer patients with a stoma, of whom 214 (64.4%) exhibited BID (BIS ≥ 10). These findings are similar to those reported by Phung et al. (16) but higher than those reported by Song et al. (10). Previous qualitative research (9) has also shown that patients with a stoma commonly experience BID accompanied by decreased self-confidence. The LPA method is commonly employed to identify latent profiles (31). This study identified three distinct categories: low body image disturbance-emotional stability group, moderate body image disturbance-cognitive regulation group, and high body image disturbance-social avoidance group. Individuals in low body image disturbance-emotional stability group demonstrated strong adaptability to bodily changes but may still exhibit mild tendencies toward social avoidance in specific situations; nursing staff should strengthen follow-up care to encourage patients to maintain active social participation. Patients in the moderate body image disturbance-cognitive regulation group exhibit moderate impairment in BI but retain certain cognitive regulation and adaptive abilities when coping with bodily changes. We recommend that healthcare providers develop targeted health education and cognitive-behavioral intervention strategies to facilitate a transition from “cognitive regulation” to “good adaptation” and prevent progression to high levels of BID. Patients in the high body image disturbance-social avoidance group exhibited pronounced social avoidance behaviors. Such patients are prone to feelings of dissatisfaction and aversion toward changes in body structure and perceive the external environment as threatening, thereby reducing social interaction as a means of emotional buffering and self-defense (32). Therefore, healthcare providers should pay attention to these patients’ social avoidance behaviors and improve their BI through measures such as promoting social participation and reducing avoidance tendencies.

### 4.2 Factors associated with latent profiles of BI

#### 4.2.1 Effects of sex, age, place of residence, and educational level on BI

This study indicates that male patients had lower odds of being classified into the high body image disturbance-social avoidance group (OR = 0.178), and that female patients with a stoma exhibited poorer BI, consistent with the findings of Liu et al. (33). The findings may be related to the influence of traditional Chinese values, under which men bear multiple social roles and family responsibilities and are typically expected to exhibit traits of strength and self-reliance. Consequently, when faced with changes in physical function or appearance, they may choose to suppress or ignore their emotional experiences to avoid revealing vulnerability or negative emotions. Therefore, healthcare providers should help male patients recognize the importance of seeking emotional support and offer training in emotional management and expression to alleviate BID that may be exacerbated by gender-related constraints.

The findings of this study indicate that colorectal cancer patients with a stoma aged under 50 are more likely to be classified into the high body image disturbance-social avoidance group (OR = 8.391). These findings are consistent with those reported by Yang et al. (34). Young patients with a stoma are in the prime of their lives when they develop the condition; they have high standards regarding their appearance, and carrying a stoma bag while working outside the home is inconvenient. Additionally, they may have relatively limited experience coping with major life stressors, and the diagnosis of cancer and the creation of a stoma often constitute a significant psychological shock for them. Therefore, healthcare providers should implement stratified management based on age differences, focusing particularly on younger stoma patients, and regularly organize patient communication sessions to promote psychological adaptation and the restoration of social functioning.

Our findings indicate that patients who have lived in rural areas for a long time are more likely to be classified into the moderate body image disturbance-cognitive regulation group and high body image disturbance-social avoidance group (OR = 3.429, OR = 4.429). The relative scarcity of medical resources in rural areas makes it difficult for patients to access psychological support and health education (35). In rural areas, illnesses and stoma surgeries are more likely to be associated with social stigma, causing patients to experience feelings of aversion and dissatisfaction, as well as anxiety about being rejected or discriminated against due to their stoma. Healthcare professionals can utilize telemedicine technologies to provide psychological support and health education to rural patients, thereby reducing their levels of BID.

This finding indicates that patients with a junior high school education are more likely to be classified into the moderate body image disturbance-cognitive regulation group and the high body image disturbance-social avoidance group (OR = 4.089, OR = 12.885); however, these results contradict those of another study (36). Previous research (37) has indicated that educational level can significantly influence sexual function and appearance-modifying behaviors in stoma patients. Patients with lower educational attainment may have limited access to disease-related information and coping resources. Although they may possess a basic awareness of their condition, their understanding is often not supported by sufficient professional medical knowledge, which may increase uncertainty and misconceptions about the disease. Additionally, differences in geographic location and cultural background may contribute to variations in research findings. Healthcare providers should provide visual and individualized health education to improve patients’ understanding of and ability to manage their condition, strengthen emotional support and communication, and alleviate disease-related fear and anxiety, thereby promoting improvements in BI.

#### 4.2.2 The effect of time since stoma creation and stoma complications on BI

Our results indicate that patients who underwent stoma surgery within the past 3 months were more likely to be classified into the moderate body image disturbance-cognitive regulation group and high body image disturbance-social avoidance group (OR = 4.995, OR = 3.302). This is consistent with the findings of Wang et al. (38), suggesting that changes in physical appearance and functional restructuring following stoma surgery make it difficult for patients to accept the stoma in the short term. These findings suggest that healthcare providers should inform patients in advance about the impacts of changes in bowel habits, provide thorough postoperative health education, and guide patients on the proper use of stoma bags to minimize adverse experiences such as leakage and odor. This approach enables patients to adapt to the effects of the stoma as early as possible after surgery, thereby reducing social concerns and improving their postoperative quality of life.

This finding indicates that patients with stoma complications are more likely to be classified into the high body image disturbance-social avoidance group (OR = 5.881). Although only 35.2% of patients in the present study experienced stoma complications, previous studies have reported that the incidence of stoma complications may reach 70% in some populations (39). The functional impairments, physical discomfort, and appearance concerns caused by stoma complications further reinforce patients’ negative self-image and BID, thereby intensifying social avoidance behaviors (40). Research indicates (41) that the pathological appearance associated with stoma complications contributes to stoma patients’ self-loathing and rejection of their own bodies, leading to a negative BI. Therefore, healthcare professionals should enhance patients’ stoma care skills and strengthen health education focused on preventing stoma complications.

#### 4.2.3 The effects of social support, anxiety and depression, stoma adaptation, and perceived illness on the BI

The findings indicate that perceived social support was negatively associated with membership in the high body image disturbance-social avoidance group (OR = 0.941), and patients with higher anxiety and depression scores were more likely to be classified into the high body image disturbance-social avoidance group (OR = 1.185). Lower levels of stoma adaptation were associated with a higher likelihood of being classified as moderate body image disturbance-cognitive regulation group and high body image disturbance-social avoidance group (OR = 0.955, OR = 0.867), while patients with higher illness perception scores were more likely to be classified as moderate body image disturbance-cognitive regulation group and high body image disturbance-social avoidance group (OR = 1.043, OR = 1.058). This suggests that differences in BI among colorectal cancer patients with a stoma are not solely due to changes in physical appearance but result from the combined effects of multiple psychosocial factors. Social support plays a crucial role in patients’ coping with the disease; patients with insufficient social support are more likely to feel external pressure or discrimination, experience impaired self-identity, and consequently reduce social activities and avoid interpersonal interactions (42).

Perception of illness refers to an individual’s subjective understanding of the disease and serves as the starting point for illness cognition. When patients perceive the changes brought about by the stoma as uncontrollable and significantly impacting their lives, they are likely to view it as a burden, leading to negative perceptions (43). Anxiety and depression, as emotional responses, can further amplify this burden, making patients more prone to developing BID through avoidance behaviors (44). In contrast, patients with higher levels of stoma adaptation gradually accept the stoma in their daily care practices. They can independently change stoma bags, manage leakage, and attempt to go out, thereby reducing negative perceptions (45) and improving BI. These findings indicate that in clinical practice, interventions should not be limited to stoma care itself but should also address patients’ cognition, emotions, social support, and adaptive capacity. Healthcare professionals should actively conduct health education to help patients better understand the stoma and postoperative changes, thereby reducing postoperative anxiety. At the same time, they should prioritize the identification and management of anxiety and depression, and encourage family members to participate in care and provide support to enhance patients’ sense of social support. Additionally, healthcare professionals should strengthen guidance on stoma self-care skills for patients, thereby improving their adaptive capacity and confidence and gradually enhancing their BI.

## 5 Limitations

Firstly, the sample was drawn exclusively from a single Grade A tertiary hospital in China. Other regions and hospital types may differ in terms of culture, economic status, and quality of care; therefore, future studies will adopt a multicenter, large-sample design. Secondly, this study employed a cross-sectional design, and the effect of stoma type on BI was not statistically significant (*p* > 0.05). In future studies, a longitudinal design could be used to group patients by stoma type for analysis, thereby providing a deeper understanding of the characteristics and differences in their BI changes.

## 6 Conclusion

This study found that BI levels vary among colorectal cancer patients with a stoma, identifying three BI groups: low body image disturbance-emotional stability, moderate body image disturbance-cognitive regulation, and high body image disturbance-social avoidance. We further identified age, gender, place of residence, educational level, stoma complications, time since stoma creation, social support, anxiety and depression, stoma adaptation, and illness perception as factors influencing the latent profiles of BI in colorectal cancer patients with a stoma. This study provides evidence for the early identification of different BI profiles among patients and developing targeted nursing intervention strategies.

## Data Availability

The raw data supporting the conclusions of this article will be made available by the authors, without undue reservation.
