# Supplementary material for: Body image and its influencing factors in colorectal cancer patients with a stoma: a latent profile analysis
Source: Front Public Health. 2026 Jun 8;14:1860267. doi: 10.3389/fpubh.2026.1860267 (PMC13307300; doi:10.3389/fpubh.2026.1860267)
Supplement: Supplementary file 1 [file Data_Sheet_1.DOCX]

**Questionnaire for Colorectal Cancer Patients with a Stoma**

**Regarding Body Image**

Name Department Diagnosis

Medical Record Number Telephone

****D**ear Participant,**

This study focuses on body image among colorectal cancer patients with a stoma. Previous studies have shown that patients with a stoma may experience varying degrees of body image changes after surgery, which may affect their psychological well-being and quality of life. This study aims to explore different patterns of body image changes and the factors associated with them, in order to provide evidence for more targeted nursing and psychological support in the future.

In this study, we will collect information related to your current condition, including questionnaire responses and relevant clinical information. The findings may help healthcare professionals better understand body image changes among patients with a stoma and improve supportive care.

This study only involves completing questionnaires and collecting relevant medical information. No additional examinations or treatments will be required, and participation will not result in any extra medical costs or affect your current or future medical care. All information you provide will be kept strictly confidential and used for research purposes only.

Your participation is entirely voluntary. You are free to withdraw from the study at any time without any impact on your medical care. If you have read and understood the information above and agree to participate, you may choose to participate in this study.

Thank you very much for your time and support.

Participant Signature:

Investigator:

Survey Date:

1. **Demographic information and clinical characteristics questionnaire**

Age: _______ years

Sex:
① Male  ② Female

Monthly household income per capita: _______ RMB

Place of residence:
① Urban
② Rural
③ Other

Education level:
① Primary school or below
② Junior high school / technical secondary school
③ High school or college and above

II. Clinical Information

Tumor stage:
① Stage I ② Stage II ③ Stage III ④ Stage IV

Time since surgery: _______

Adjuvant therapy:
① Yes  ② No

Type of stoma:
① Temporary stoma
② Permanent stoma

Stoma-related complications:
① Yes  ② No

1. **Body Image Scale (BIS)**

Please indicate how you have felt about your appearance over the past week, including any changes caused by your illness or treatment. Tick (√) the option that best applies to you.

|  | Item | Not at all | A little | Quite a bit | Very much |
| --- | --- | --- | --- | --- | --- |
| 1 | Feeling self-conscious about appearance | 0 | 1 | 2 | 3 |
| 2 | Feeling less physically attractive | 0 | 1 | 2 | 3 |
| 3 | Dissatisfied with physical appearance | 0 | 1 | 2 | 3 |
| 4 | Feeling less feminine or masculine | 0 | 1 | 2 | 3 |
| 5 | Difficulty looking at yourself when undressed | 0 | 1 | 2 | 3 |
| 6 | Feeling less sexually attractive | 0 | 1 | 2 | 3 |
| 7 | Avoiding crowds | 0 | 1 | 2 | 3 |
| 8 | Feeling physically incomplete | 0 | 1 | 2 | 3 |
| 9 | Dissatisfied with one’s body | 0 | 1 | 2 | 3 |
| 10 | Feeling dissatisfied with scars | 0 | 1 | 2 | 3 |

1. **Perceived Social Support Scale (PSSS)**

The following are 12 statements, each with 7 response options. Please select one answer for each statement based on your actual situation.

| Item | Very strongly disagree | Strongly disagree | Mildly disagree | Neutral | Mildly agree | Strongly agree | Very strongly agree |
| --- | --- | --- | --- | --- | --- | --- | --- |
| 1. My family really tries to help me. | 1 | 2 | 3 | 4 | 5 | 6 | 7 |
| 1. I can talk about my problems with my family. | 1 | 2 | 3 | 4 | 5 | 6 | 7 |
| 1. My family is willing to help me make decisions. | 1 | 2 | 3 | 4 | 5 | 6 | 7 |
| 1. I get the emotional help and support I need from my family. | 1 | 2 | 3 | 4 | 5 | 6 | 7 |
| 1. My friends really try to help me. | 1 | 2 | 3 | 4 | 5 | 6 | 7 |
| 1. I can share my joys and sorrows with my friends. | 1 | 2 | 3 | 4 | 5 | 6 | 7 |
| 1. I can count on my friends when things go wrong. | 1 | 2 | 3 | 4 | 5 | 6 | 7 |
| 1. I can talk about my problems with my friends. | 1 | 2 | 3 | 4 | 5 | 6 | 7 |
| 1. There are some people (e.g., leaders, relatives, colleagues) who are a real source of comfort to me when I am in trouble. | 1 | 2 | 3 | 4 | 5 | 6 | 7 |
| 1. There are some people (e.g., leaders, relatives, colleagues) who care about my feelings. | 1 | 2 | 3 | 4 | 5 | 6 | 7 |
| 1. There are some people (e.g., leaders, relatives, colleagues) who are around when I am in need. | 1 | 2 | 3 | 4 | 5 | 6 | 7 |
| 1. I can share my joys and sorrows with some people (e.g., leaders, relatives, colleagues). | 1 | 2 | 3 | 4 | 5 | 6 | 7 |

1. **Hospital Anxiety and Depression Scale (HADS)**

This scale consists of two subscales: anxiety and depression, each containing 7 items. Please select the option that best describes your situation for each statement.

1. I feel tense or “wound up”.

0=Not at all

1=Sometimes

2=Often

3=Most of the time

1. I get a sort of frightened feeling as if something awful is about to happen.

0=Not at all

1=A little, but it doesn’t worry me

2=Yes, but not too badly

3=Very definitely and quite badly

1. Worrying thoughts go through my mind.

0=Only occasionally

1=Sometimes, but not too badly

2=Often

3=Most of the time

1. I can sit at ease and feel relaxed.

0=Definitely

1=Usually

2=Not often

3=Not at all

1. I get a sort of frightened feeling like “butterflies” in the stomach.

0=Not at all

1=Occasionally

2=Quite often

3=Very often

1. I get sudden feelings of panic.

0=Not at all

1=Not very often

2=Quite often

3=Very often indeed

1. I feel restless as if I have to be on the move.

0=Not at all

1=Not very much

2=Quite a lot

3=Very much indeed

1. I still enjoy the things I used to enjoy.

0=Definitely as much

1=Not quite so much

2=Only a little

3=Hardly at all

1. I can laugh and see the funny side of things.

0=As much as I always could

1=Not quite so much now

2=Definitely not so much now

3=Not at all

1. I feel cheerful.

0=Most of the time

1=Sometimes

2=Not often

3=Not at all

1. I have lost interest in my appearance.

0=I take just as much care as ever

1=I may not take quite as much care

2=I don’t take as much care as I should

3=I definitely don’t take as much care

1. I look forward with enjoyment to things.

0=As much as I ever did

1=Rather less than I used to

2=Definitely less than I used to

3=Hardly at all

1. I get a sort of slowed down feeling.

0=Not at all

1=Sometimes

2=Often

3=Nearly all the time

1. I can enjoy a good book or radio or TV program.

0=Often

1=Sometimes

2=Not often

3=Rarely

1. **Ostomy Adjustment Inventory-23**

Please select the option that best describes your current feelings and mark it with a tick (√).

| Item | Strongly agree | Agree | Uncertain | Disagree | Strongly disagree |
| --- | --- | --- | --- | --- | --- |
| 1. Because of my stoma, I feel that I will always be a patient. |  |  |  |  |  |
| 2. Taking care of my stoma is difficult. |  |  |  |  |  |
| 3. My stoma limits my activities. |  |  |  |  |  |
| 4. I often worry about leakage, odor, or noise from my stoma. |  |  |  |  |  |
| 5. I have not yet accepted my stoma. |  |  |  |  |  |
| 6. I often feel anxious because of my stoma. |  |  |  |  |  |
| 7. I feel that my stoma has reduced my sexual attractiveness. |  |  |  |  |  |
| 8. Since having a stoma, I have avoided social activities. |  |  |  |  |  |
| 9. Because of my stoma, I feel that my life is out of control. |  |  |  |  |  |
| 10. I believe that I will be able to manage my stoma well in the future. |  |  |  |  |  |
| 11. Despite having a stoma, my life is still meaningful. |  |  |  |  |  |
| 12. Even after stoma surgery, I still feel that life is meaningful. |  |  |  |  |  |
| 13. I have come to regard my stoma as part of my body. |  |  |  |  |  |
| 14. Despite having a stoma, I am still able to participate in various activities. |  |  |  |  |  |
| 15. I feel grateful, as my stoma has given me a new life. |  |  |  |  |  |
| 16. After having a stoma, I feel that my life is no longer threatened by my previous illness. |  |  |  |  |  |
| 17. I am able to sleep well without worrying about my stoma. |  |  |  |  |  |
| 18. I feel that I have recovered from stoma surgery. |  |  |  |  |  |
| 19. My appetite is as good as it was before the stoma surgery. |  |  |  |  |  |
| 20. I have accepted the changes in my appearance caused by the stoma. |  |  |  |  |  |

1. **Brief Illness Perception Questionnaire (B-IPQ)**

Please tick (√) the number that best describes your views about your illness.

1. How much does your illness affect your life?

0 1 2 3 4 5 6 7 8 9 10

No effect at all Severely affects my life

1. How long do you think your illness will last?

0 1 2 3 4 5 6 7 8 9 10

A very short time A very long time

1. How much control do you feel you have over your illness?

0 1 2 3 4 5 6 7 8 9 10

No control at all A great deal of control

1. How much do you think your treatment can help your illness?

0 1 2 3 4 5 6 7 8 9 10

Not helpful at all Extremely helpful

1. How much do you experience symptoms from your illness?

0 1 2 3 4 5 6 7 8 9 10

No symptoms at all Severe symptoms

1. How concerned are you about your illness?

0 1 2 3 4 5 6 7 8 9 10

Not at all concerned Extremely concerned

1. How well do you feel you understand your illness?

0 1 2 3 4 5 6 7 8 9 10

Do not understand at all Understand my illness very clearly

1. How much does your illness affect you emotionally (e.g., make you feel angry, afraid, upset, or depressed)?

0 1 2 3 4 5 6 7 8 9 10

Not affected emotionally at all Extremely affected emotionally
